# Calein C, a Sesquiterpene Lactone Isolated From *Calea Pinnatifida* (*Asteraceae*), Inhibits Mitotic Progression and Induces Apoptosis in MCF-7 Cells

**DOI:** 10.3389/fphar.2018.01191

**Published:** 2018-10-18

**Authors:** Lhaís Araújo Caldas, Renato O. Horvath, Guilherme Álvaro Ferreira-Silva, Marcelo J. P. Ferreira, Marisa Ionta, Patricia Sartorelli

**Affiliations:** ^1^Departamento de Química, Instituto de Ciências Ambientais, Químicas e Farmacêuticas, Universidade Federal de São Paulo, Diadema, São Paulo, Brazil; ^2^Departamento de Biologia Celular e do Desenvolvimento, Instituto de Ciências Biomédicas, Universidade Federal de Alfenas, Alfenas, Brazil; ^3^Departamento de Botânica, Instituto de Biociências, Universidade de São Paulo, São Paulo, Brazil

**Keywords:** Asteraceae, *Calea pinnatifida*, calein C, antimitotic activity, breast cancer, *AURKB*, *PLK-1*

## Abstract

Breast cancer is the most common cancer in women worldwide. Estrogen receptor-positive (ER+) breast cancer represents approximately 75% of diagnosed cases, while 15–20% of them are triple-negative (TN). Although there have been improvements in the therapeutic approach, the mortality rate remains elevated. Thus, it is necessary to identify new chemotherapeutic agents. The present study aimed to evaluate the effects of calein C, a sesquiterpene lactone isolated from *Calea pinnatifida*, on breast cancer cell lines MCF-7 (ER+), Hs578T (TN) and MDA-MB-231 (TN). Calein C significantly reduced the viability of all cell lines; however, MCF-7 cells were more responsive than MDA-MB-231 or Hs578T cells. Thus, the MCF-7 cell line was selected for further investigation. We demonstrated that calein C inhibited cell cycle progression in MCF-7 cells at M-phase. Increased frequency of mitosis was observed in calein C-treated samples compared to the control group, especially of the cell population in initial stages of the mitosis. These events were associated with the ability of calein C to modulate expression levels of critical regulators of mitosis progression. We observed a significant reduction in the relative mRNA abundance of *PLK1* and *AURKB* along with a concomitant increase in *CDKN1A* (p21) in treated samples. In addition, calein C induced apoptosis in MCF-7 cells due to, at least in part, its ability to reduce the *BCL2/BAX* ratio. Therefore, our data provide evidence that calein C is an important antimitotic agent and should be considered for further *in vivo* investigations.

## Background

Breast cancer (BC) is one of the most common cancers in women across the world. Despite the development of therapeutic treatments in recent decades, it remains the major cause of cancer death in women worldwide (Bray et al., 2018). BC is a heterogeneous disease characterized by a wide spectrum of clinical, pathological, and molecular features (Perou et al., 2000; Sorlie, 2004). Triple-negative BC is characterized by an absence of estrogen and progesterone receptors and a lack of the overexpression of human epidermal receptor 2 (HER2). The absence of therapeutic targets in TN breast cancer limits treatment options (Oualla et al., 2017). ER+ BC represents approximately 75% of diagnosed breast cancers, and endocrine therapy is the most commonly used treatment for patients with ER+ malignant breast tumors (Bray et al., 2018). However, 30–50% of initially responsive patients develop resistance to these endocrine treatments (Augusto et al., 2018). Thus, it is critical to identify novel compounds with antitumor activity to improve the therapeutic approaches for breast cancer patients.

The development of antineoplastic drugs in recent decades resulted from combinatory chemistry, which characterizes compounds from natural, synthetic and semisynthetic sources, as alternatives to cancer treatment (Newman and Cragg, 2016). The number of discoveries of natural compounds with anticancer properties has increased in the last several years and has become an important tool for the development of semisynthetic and synthetic drugs inspired by natural products, providing an alternative to cancer treatment and attempting to decrease side effects and improve efficacy. According to data in the literature, between 1981 and 2014, approximately 1500 new drugs were approved by the FDA, and 980 of them were natural products or derivatives (Newman and Cragg, 2016). Among the main compounds known currently, paclitaxel (isolated from *Taxus baccata* and *Taxus brevifolia*) and vinblastine isolated from (*Catharanthus roseus*) are the most used therapeutic anticancer drugs from natural products (Li and Lou, 2017).

In Asteraceae, the presence of sesquiterpene lactones, the taxonomic characteristic of the family, is related to many biological activities, such as antiparasitic, anti-inflammatory, antileishmanial, antitrypanosomal, antimicrobial and cytotoxic activities. The major compounds credited to these pharmacological effects are germacranolides and chromenes (Nakagawa et al., 2005). Previous studies have shown that the cytotoxic activities of the germacranolides present in *Calea uniflora* are effective against a leukemia cell line U937 (Yamada et al., 2004). Antileishmanial activity of chromenes isolated from *Calea pinnatifida* is effective against *Leishmania amazonensis*; and antitrypanosomal activity is effective against *Trypanosoma cruzi* (Lima et al., 2015).

The studied species, *Calea pinnatifida* (R. Br) Less (Asteraceae), is popularly known as *aruca, cipó cruz* or *quebra-tudo*. The geographical distribution of the plant in Brazil occurs mainly in the cerrado biome (Lorenzi and Matos, 2002). The species is popularly used in folk medicine as tea and infusions in order to treat stomach aches, giardiasis, amoebiasis, and gastric disorders in general (Ferreira et al., 1980). According to the literature, phytochemical studies of the components of the plant have revealed that they are mainly germacranolides, fatty esters, stigmasterols, sitosterols, and polyacetylene derivatives (Ferreira et al., 1980). Among all of the isolated compounds from *C. pinnatifida* at present, arucanolide has been shown to be the major secondary metabolite of the species and the main metabolite responsible for the cytotoxic activity of the plant. Previous studies have described the antitumor activity of crude dichloromethane extract from *Calea pinnatifida*, which results in a reduction of approximately 70% of the tumor volume (Marchetti et al., 2012). Also the same authors suggested that arucanolide, the major compound of the extract, might be responsible for this cytotoxic activity (Marchetti et al., 2010). According to Gopfert et al. (2005), arucanolide, being a sesquiterpene lactone, is usually found in the aerial parts of the plants, specifically compartmentalized in glandular trichomes as a manner to avoid autotoxicity. Thus, the present study was undertaken to evaluate the cytotoxic activity of a sesquiterpene lactone isomer of the arucanolide, with the acetate and methacrylate groups reversed at the C-8 and C-9 carbons (defined by the 2D NMR spectra), named calein C, isolated from leaves of *C. pinnatifida*, on cells derived from breast cancer. In addition, we investigated the molecular mechanism underlying the cytotoxic activity of calein C on MCF-7 cells.

## Materials and methods

### General experimental procedures

The ^1^H and ^13^C NMR uni- and bidimensional spectra of compounds were recorded at 300 and 75 MHz, respectively, in a Bruker UltraShield 300 Advance III spectrometer. CDCl_3_ and benzene-d_6_ (Aldrich) were used as the solvent and as the internal standard. Silica gel *flash* (Merck, 230–400 mesh) and Sephadex LH-20 were used for column chromatographic separation, while a 60 PF_254_ silica gel (Merck) was used for analytical and preparative TLC. HPLC analysis was performed using a Dionex Ultimate 3000 chromatograph, a Luna Phenomenex RP-18 column (3 μm, 150 × 5 mm) and a UV-DAD detector.

### Plant material

Leaves of *Calea pinnatifida* were collected from a single tree in the Atlantic Forest area of São Paulo City, SP, Brazil (coordinates 23 53′08.86″S, 46 40′10.45″W), in October 2012. A voucher specimen (C.R. Figueiredo 25) has been deposited in the SPF Herbarium of Departamento de Botânica from Instituto de Biociências of Universidade de São Paulo.

### Extraction and isolation of Calein C

Fresh leaves of *C. pinnatifida* (300 g), were dried, ground and then exhaustively extracted using MeOH at room temperature. After evaporation of the solvent under reduced pressure, the obtained crude extract (10 g) was resuspended in MeOH:H_2_O 2:1. CH_2_Cl_2_ phase (650 mg) was subjected to column chromatography (CC) over Sephadex LH-20 using MeOH as the mobile phase to give seven fractions (A–G). Fraction B (420 mg) was subjected to flash CC over silica using increasing amounts of MeOH in CH_2_Cl_2_ as solvent to afford three fractions (B1–B3). Part of this fraction (100 mg) was purified over semipreparative RP-18 HPLC and eluted with ACN:H_2_O 4:6 (flow rates 3.6 mL/min, UV 218 nm) to obtain calein C (40 mg).

### Cell lines and treatment schedule

Human breast cancer cell lines were used in the present study (MCF-7, estrogen receptor-positive; Hs578T, triple-negative; and MDA-MB-231, triple-negative). CCD-1059Sk, a normal cell line (fibroblast derived from human skin), was also examined. The cell lines used were purchased from the Rio de Janeiro Cell Bank. Cell cultures were maintained in DMEM (Dulbecco's modified Eagle's medium, Sigma, CA, USA) supplemented with 10% fetal bovine serum (Vitrocell, Campinas, Brazil). Cells were grown in a humidified atmosphere of 95% air and 5% CO_2_ at 37°C. After attachment (24 h), the cells were treated for 24 or 48 h according to the experimental approach.

### Cell viability analysis

Cell viability was measured by MTS (dimethylthiazol carboxymethoxyphenyl sulfophenyl tetrazolium) assay using the CellTiter 96® Aqueous Non-Radiative Cell Proliferation assay (Promega) according to the manufacturer's instructions. Formazan, the reduced form of tetrazolium, absorbs light at 490 nm and viability rate is directly proportional to the amount of formazan produced by dehydrogenase enzymes. Cells were seeded into a 96-well plate at 1 × 10^4^ cells/well. After attachment, the cells were treated with calein C at different concentrations for 24 h or 48 h. Experiments were conducted in triplicate. Data are presented as the mean ± standard deviation (SD) of three independent experiments. The IC_50_ value was determined from nonlinear regression using GraphPad Prism® (GraphPad Software, Inc., San Diego, CA, USA).

### Clonogenic assay

The clonogenic assay was performed according to Franken et al. (2006). Briefly, 200 cells were seeded into 35 mm plates. Cells were treated for 24 h and recovered in drug-free medium for the subsequent 14 days. Afterwards, the colonies were fixed and stained with crystal violet. Only colonies with >50 cells were counted by direct visual inspection with a stereomicroscope at 20x magnification. The assays were performed in triplicate, and the data were presented as the mean ± SD of three independent experiments.

### Cell cycle analysis

Cell cycle analysis was performed according to Ferreira-Silva et al. (2017). Briefly, cells were treated with calein C for 24 h at 7.5 and 15.0 μg mL^−1^. Cells were fixed with ethanol (75% in PBS, phosphate-buffered saline) at 4°C overnight. Afterwards, the cells were homogenized in a dye solution [PBS containing 300 μg mL^−1^ propidium iodide (PI) and 5 mg mL^−1^ RNAse]. DNA was quantified 1 h after staining. The analysis was performed by flow cytometry (Guava easyCyte 8HT, Hayward, CA, USA). The results are presented as the mean ± SD of three independent experiments.

### Apoptosis evaluation using Annexin V assay

Cells were seeded into 24-well plates at 5 × 10^4^ cells/well. After 24 h of treatment with calein C at a concentration of 7.5 or 15.0 μg mL^−1^, we evaluated the phosphatidylserine externalization using the Guava Nexin® Kit (Merck Millipore, Massachusetts, USA) according to manufacturer's instructions. Briefly, cells were collected by enzymatic digestion (Trypsin/EDTA, Sigma), centrifuged at 1,000 rpm for 5 min at 4°C, washed with ice-cold PBS, and then 2 × 10^4^ cells were resuspended in 100 μL of DMEM. In the next step, 100 μL of a mixed solution containing buffered Annexin V-PE and 7-AAD was added to the cells. The samples were read after 20 min of incubation at room temperature in a dark chamber. Carbonyl cyanide *m*-chlorophenylhydrazone (CCCP) (Sigma-Aldrich) at 25 μM was used as a positive control as it induces mitochondrial membrane depolarization. The analysis was performed by flow cytometry using GuavaSoft 2.7 software. The experiments were conducted in triplicate and repeated twice. The data are presented as the mean ± SD.

### Immunofluorescence and mitotic index

Cells were seeded into 35 mm Petri plates on coverslips at 2 × 10^5^ cells/plate. After treatment, the samples were fixed with 3.7% formaldehyde for 30 min. For α-tubulin immunolabeling, cells were permeabilized with Triton X-100 (0.5%) for 10 min. After blocking with 1% BSA, anti-α-tubulin antibody (1:100, clone DM1A, Sigma Aldrich) was incubated overnight. On the next day, secondary anti-mouse IgG-FITC antibody (1:100, Sigma Aldrich) was added to the sample and incubated for 2 h. Then, labeling with Phalloidin–TRITC (Sigma Aldrich) was performed for an additional 1 h. Nuclei were stained with DAPI, and the coverslips were mounted on microscope slides using Vecta-Shield mounting medium (Vector Laboratories). Analyses were performed using a fluorescence microscope (Nikon). In addition, the fluorescent cytological preparations were used to determine the frequency of mitosis. 1,000 cells per sample were counted. The data are shown as the mean ± SD of four independent experiments.

### Immunoblotting

Cells were homogenized in RIPA lysis buffer (150 mM NaCl, 1.0% Nonidet P-40, 0.5% deoxycholate, 0.1% SDS and 50 mM Tris pH 8.0) containing both protease and phosphatase inhibitors (Sigma). Lysates were centrifuged (12,000 × g) for 5 min at 4°C. Supernatants were recovered, and total proteins were quantified (BCA kit, Pierce Biotechnology Inc., Rockford, IL, USA) and resuspended in Laemmli sample buffer containing 62.5 mM Tris–HCl pH 6.8, 2% SDS, 10% glycerol, 5% 2-mercaptoethanol and 0.001% bromophenol blue. An aliquot of 30 μg of protein was separated by SDS–PAGE (12%), transferred (100 V, 250 mA for 2 h) onto a PVDF membrane (Amersham Bioscience), and blocked for 1 h at 4°C with blocking solution [5% nonfat milk in Tris-buffered saline (TBS) + 0.1% (v/v) Tween-20] to prevent nonspecific protein binding. The membrane was probed overnight at 4°C with the following primary antibodies: cyclin B1 (SC-245, Santa Cruz – 1: 200), phospho-Histone H3 (Ser10) (SC- 8656-R, Santa Cruz - 1:200), and α-tubulin (clone DM1A, Sigma– 1:2,000). After washing with TBS-tween (0.1%), the membrane was incubated with a secondary antibody (anti-rabbit or anti-mouse peroxidase conjugated) for 2 h at room temperature. Immunoreactive bands were visualized with the ECL Western Blotting Detection Kit (Amersham Pharmacia). A reprobing protocol was followed for detecting immunoreactive bands using the different antibodies. The results were obtained from three independent experiments. The quantification of immunoreactive bands was performed using a public program (ImageJ).

### Transcript level evaluation

Total RNA from the treated and untreated MCF-7 cells was extracted using the RNeasy® Mini kit (Qiagen, Mississauga, ON, Canada), according to the manufacturer's instructions, and was eluted in 30 μL of RNAse-free water. RNA concentrations were measured using a spectrophotometer using a NanoDrop® ND 1000 (Thermo Scientific, Wilmington, DE, USA) and 1 μg of total RNA was incubated with DNase I (1 U/μg; Invitrogen, São Paulo, SP, Brazil) to eliminate possible contamination with genomic DNA, and then subjected to reverse transcription using random primers and the High Capacity cDNA Reverse Transcription Kit® (Applied Biosystems, São Paulo, SP, Brazil), according to the manufacturer's instructions. The reagents were incubated at 25°C for 10 min, 37°C for 120 min, and finally 85°C for 5 min to inactivate the enzyme.

Expression of the target genes (*CDKN1A, PLK1*, and *AURKB*) was investigated by real-time PCR with an ABI 7500 thermocycler using the Power Sybr Green PCR Master Mix (Applied Biosystems) and the primers as stated in Table 1. Reactions were performed in a final volume of 25 μL and the genes were amplified using the following conditions: 95°C for 10 min (1 cycle), denaturation at 95°C for 10 s, and annealing and extension at 60°C for 1 min (40 cycles). To select the most stable housekeeping gene, the amplification profiles of β-actin (*ACTB*), glyceraldehyde-3-phosphate dehydrogenase (*GAPDH*), and 18S ribosomal RNA (*18srRNA*) were compared using the geNorm applet for Microsoft Excel (medgen.ugent.be/genorm; Vandesompele et al. (2002); the most stable housekeeping gene was *ATCB*. The relative abundance of each target gene was calculated using the ΔΔCt method with an efficiency correction and a control sample for calibration (Pfaffl, 2001). The average efficiency values for each gene were calculated with the amplification profile of each sample using the LinRegPCR program (Ramakers et al., 2003). Each sample was run in triplicate, and a nontemplate control was included. The data are presented as the mean ± standard error of the mean (SEM) from 3 independent experiments.

**Table 1:** Information for specific primers used for amplification in real-time PCR.

**Gene**	**Sequence**	**References**
*CDKN1A*	F 5′-CCATAGCCTCTACTGCCACCATC-3′	NM_001291549.1
	R 5′-GTCCAGCGACCTTCCTCATCCA-3′	
*PLK1*	F 5′-CCTGCACCGAAACCGAGTTAT-3′	NM_005030.5
	R 5′-CCGTCATATTCGACTTTGGTTGC-3′	
*Aurora-B*	F 5′-AAAGAGCCTGTCACCCCATC-3′	XM_017025310.1
	R 5′-CGCCCAATCTCAAAGTCATC-3′	
*ACTB*	F 5′-AGAGCTACGAGCTGCCTGAC-3′	NM_001101.3
	R 5′-AGCACTGTGTTGGCGTACAG-3′	
*GAPDH*	F 5′-GGATTTGGTCGTATTGGGC-3′	NM_002046.4
	R 5′-TGGAAGATGGTGATGGGATT-3′	
*18srRNA*	F 5′-GTAACCCGTTGAACCCCATT-3′	HQ387008.1
	R 5′-CCATCCAATCGGTAGTAGCG-3′	

*F, forward primer; R, reverse primer*.

### Statistical analysis

The results were tested for significance using a one-way analysis of variance (ANOVA) followed by Tukey's post-test using GraphPad Prism®. The values were expressed as the mean ± SD.

## Results

### Structural characterization of Calein C

**Figure 1:**
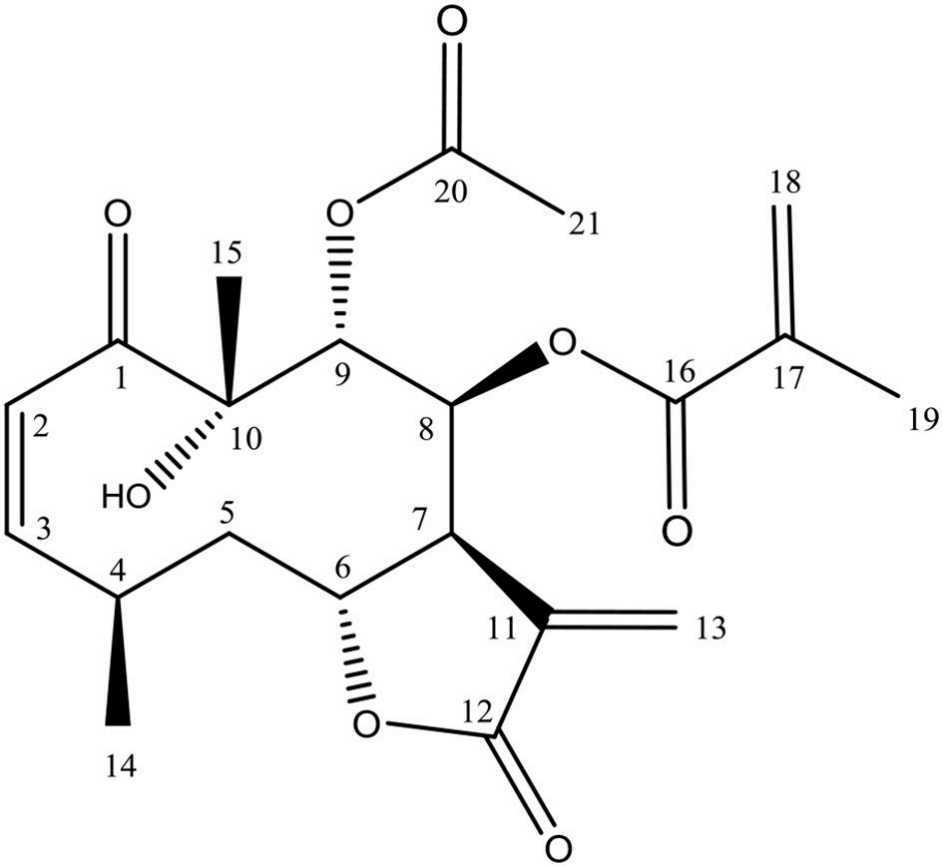
Calein C chemical structure isolated from *Calea pinnatifida*.

The chromatographic procedures carried out to active fraction B allowed the isolation of one compound. LC-HRMS-ESI (positive mode) of this compound displayed a quasi-molecular ion peak at m/z 429.1542 [M+Na]^+^, which corresponded to the formula C_21_H_26_O_8_Na (calc. m/z 429.1553), with nine unsaturations. The isolated compound was obtained as a colorless crystal, mp. 170°. ^1^H NMR (CDCl_3_, 300 MHz) δ 6.61 (*d*, 1H, *J* 12.3, H-2), 6.01 (*dd*, 1H, *J* 12.3; 12.0 Hz H-3), 3.14 (*m*, 1H, H-4), 1.45 (*m*, 1H, H-5), m, 1.83 H-5), 4.60 (*dd* 1H, *J* 5.0; 11.9, H-6), 2.64 (*br s*,1H, H-7), 5.61 (*s*, 1H, H-8), 5.62 (s, 1H, H-9), 5.84 (*d*, 1H, *J* 1.1, H-13a) 6.32 (*d*, 1H, *J* 1.1, H-13b), 1.14 (*d*, 1H, *J* 6.3, H-14), 1.35 (s, 1H, H-15), 5.55 (t, 1H, *J* 1.5, H-18), 6.02 (s, 1H, H-18b), 1.83 (s, 1H, H-19), 2.02 (s, 1H, H-21). ^13^C NMR (CDCl_3_, 75 MHz) δ 204.7 (C-1), 125.3 (C-2), 148.2 (C-3), 28.3 (C-4), 40.3 (C-5), 76.4 (C-6), 41.2 (C-7), 74.0 (C-8), 74.4 (C-9), 79.2 (C-10), 134.5 (C-11), 168.8 (C-12), 126.6 (C-13), 19.7 (C-14), 23.6 (C-15), 165.4 (C-16), 134.9 (C-17), 127.2 (C-18), 18.1 (C-19), 170.3 (C-20), 20.4 (C-21). Based on these data, it was proposed that this substance has the structure of calein C (Figure 1). The spectroscopic data can be found in Supplementary File S1.

### Biological assays

**Figure 2:**
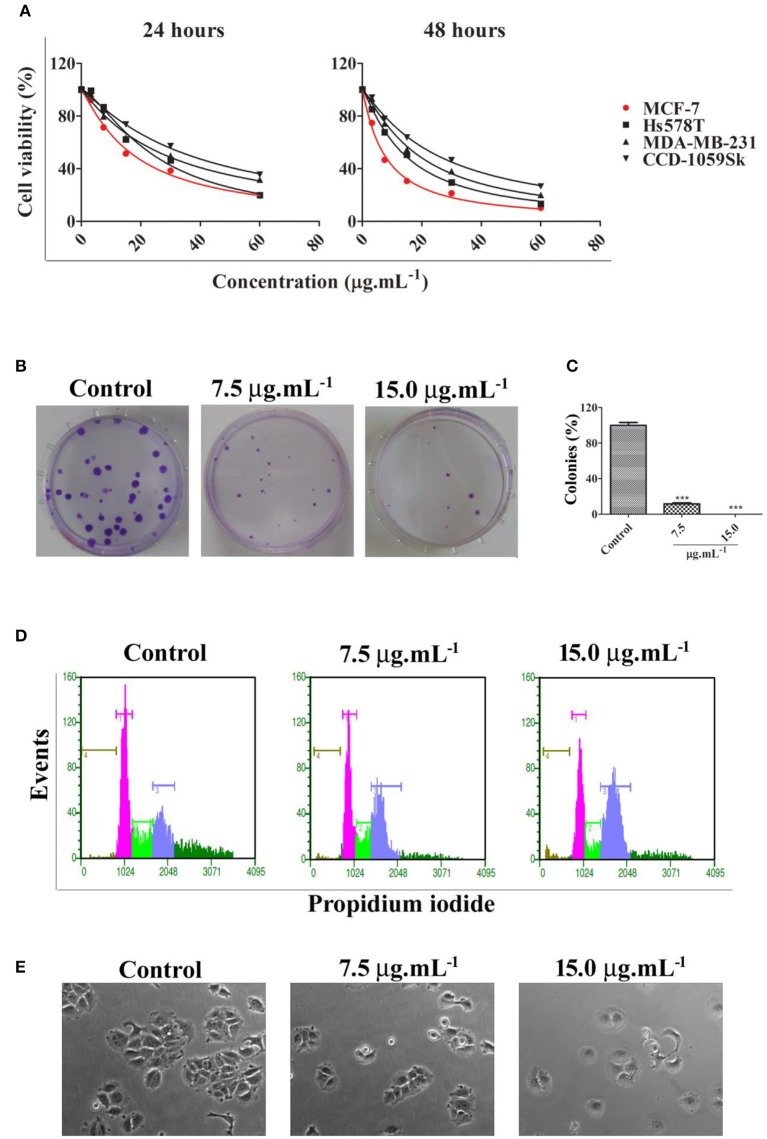
**(A)** Cell viability determined by MTS assay after 24 h and 48 h of treatment with calein C at different concentrations. MCF-7 was the most responsive cell line among the breast cancer cell lines tested (red curve). **(B)** Illustrative images from clonogenic assays. MCF-7 cells were treated for 24 h and recovered in fresh medium for an additional 14 days; **(C)** Clonogenic assay analysis. **(D)** Representative histograms obtained by flow cytometry after 24 h of treatment showing cell populations in different phases of the cell cycle: brown (sub-G1 phase), pink (G0/G1 phases), green (S phase), and blue (G2/M) phases. **(E)** Illustrative images obtained by phase contrast microscopy showing morphological features of MCF-7 cultures after 24 h of treatment (60× magnification). ***p < 0.001 according to ANOVA followed by Tukey's post-test.

Calein C effectively reduced cell viability in all cell lines studied and its effect was time- and dose-dependent; however, MCF-7 cells were more sensitive to calein C-treatment than MDA-MB-231 or Hs578T cells (Figure 2A and Table 2). In addition, when we compared the cytotoxic profiles of calein C-treated MCF-7 cells and normal cells (CCD-1059Sk), we observed that calein C had a higher selectivity toward MCF-7 cells (Figure 2A and Table 2). Therefore, MCF-7 cells were selected for further investigations.

**Table 2:** IC_50_ ± SD (μg.mL^−1^) values determined from MTS data.

	**24 h**	**48 h**	SI^*^
MCF-7	17.78 ± 0.69	7.55 ± 0.30	3.34
MDA-MB-231	28.49 ± 1.20	18.90 ± 0.55	1.33
Hs578T	24.49 ± 0.97	14.42 ± 0.28	1.75
CCD-1059Sk	36.48 ± 1.42	25.23 ± 0.63	–

* *SI (selectivity index) determined considering IC_50_ values of normal cells (CCD-1059Sk) and tumor cells ratio at 48 h treatment*.

We evaluated the potential of calein C to inhibit cell proliferation for prolonged period through clonogenic assay. The results showed a drastic reduction in the clonogenic capacity of MCF-7 cells treated with calein C once the number of colonies was significantly lower in treated samples compared to controls (Figures 2B,C). Moreover, the diameter of the colonies was smaller in treated samples when compared to the control group (Figure 2B).

**Figure 3:**
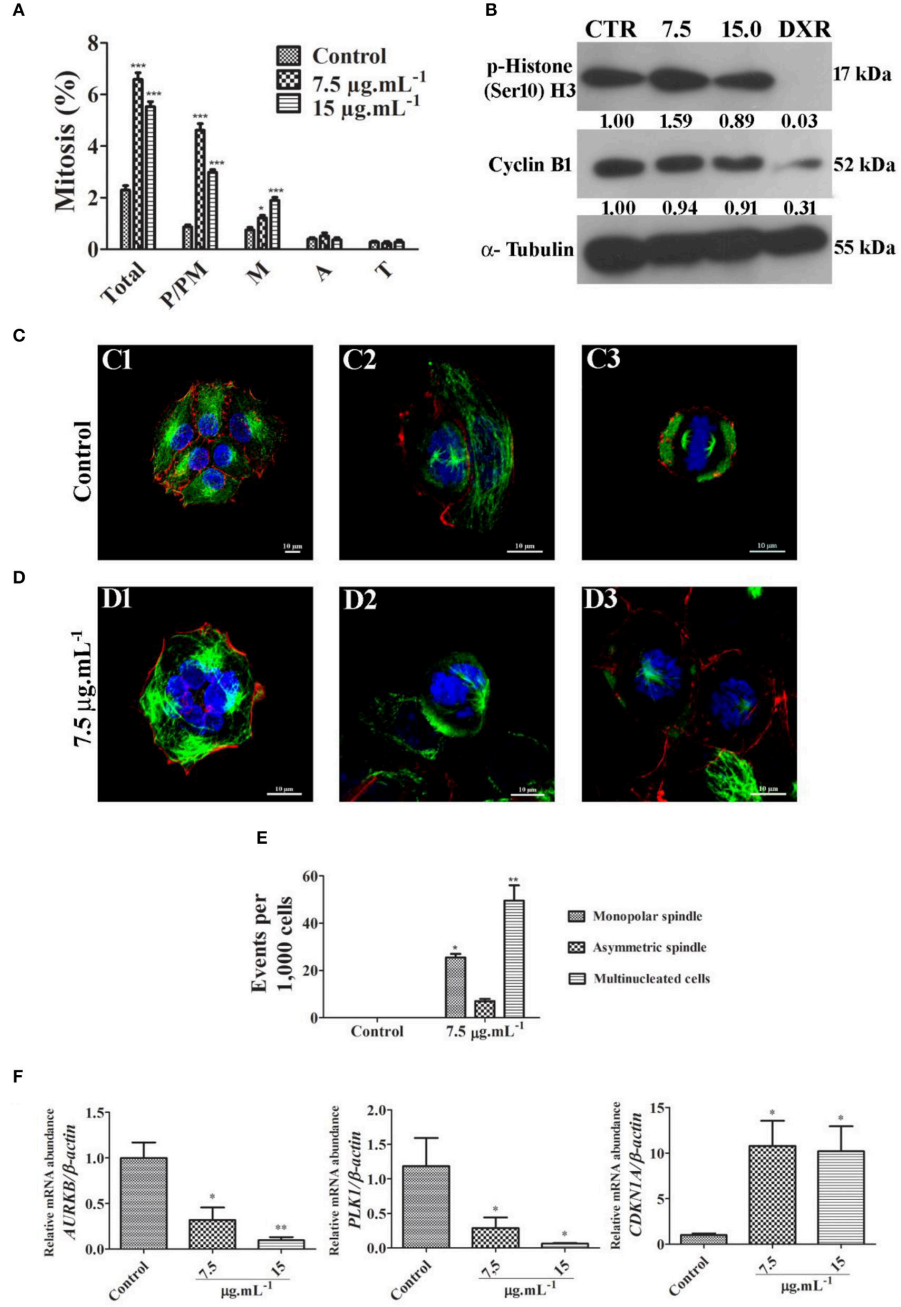
**(A)** Quantitative analysis of mitotic frequency determined from fluorescent cytological preparation labeling for microtubules, microfilaments and DNA. Total mitosis and mitosis subphases were quantified (P/PM, prophase and prometaphase; M, metaphase; A, anaphase; and T, telophase). Data represent mean ± SD from 3 independent experiments. *p < 0.05 and ***p < 0.001 according to ANOVA followed by Tukey's post-test. **(B)** Representative image of immunoblots showing the expression profile of p-Histone H3 (Ser10) and cyclin B1. Antineoplastic drug doxorubicin (DXR) was used as a positive control. **(C)** Fluorescent images obtained from control culture through confocal microscopy with a z-series projection. Note typical microtubule networks (green) in interphasic cells and bipolar mitotic spindles in mitotic cells. Microfilaments distribution pattern (red) are also shown. DNA was labeled with DAPI (blue). **(D)** Fluorescent images obtained from treated culture through confocal microscopy with a z-series projection. Multinucleated cell is shown in D1, and monopolar spindles are visualized in D2 and D3. **(E)** Quantitative analysis of frequent events observed in treated cultures. Data represent mean ± SD from 3 independent experiments. **(F)** Relative mRNA abundance of critical regulators of cell cycle determined by real-time PCR after 24 h of treatment using β*-actin* as a housekeeping gene for normalization. Data represent mean ± SEM from 4 independent experiments. *p < 0.05 and **p < 0.01 according to ANOVA followed by Tukey's post-test.

Cell cycle progression was assessed by DNA quantification after 24 h of treatment with calein C. We observed an increased G2/M population with concomitant reduction of both G0/G1 and S populations in MCF-7 cultures treated with calein C compared to the control group (Figure 2D and Table 3). In addition, a significant increase in the sub-G1 population (dead cells with fragmented DNA) was observed in samples treated with calein C at 15 μg.mL^−1^ (Figure 2D and Table 3). We also observed drastic morphological alterations in MCF-7 cells treated with calein C at 15 μg.mL^−1^ for 24 h. However, in cultures treated with the compound at 7.5 μg mL^−1^, we observed many refringent and rounded cells, which probably were in mitosis (Figure 2E). In the next step, we observed a significant increase in the frequency of mitosis in treated samples compared to the control group. Interestingly, we observed an increased frequency of cells in prophase/prometaphase and metaphase (Figure 3A). Immunoblot analysis revealed a significant increase in p-Histone H3 (Ser10) expression in samples treated with calein C at 7.5 μg.mL^−1^ for 24 h compared to the control group, reinforcing our evidence that calein C induces mitosis arrest. We also evaluated cyclin B1 expression levels, but we did not observe any alterations in the expression profile of this cyclin in treated samples when compared to the control groups (Figure 3B).

**Table 3:** Cell cycle analysis performed after 48 h of treatment with calein C.

	**DMSO**	**7.5 μg.mL^−1^**	**15.0 μg.mL^−1^**
Sub-G1	1.14 ± 0.21	1.21 ± 0.23	3.00 ± 0.24^***^
G0/G1	51.50 ± 1.00	42.69 ± 1.07^***^	33.55 ± 0.15^***^
S	18.79 ± 0.39	13.33 ± 0.45^**^	7.93 ± 1.99^***^
G2/M	28.57 ± 0.77	42.77 ± 1.24^***^	55.53 ± 2.06^***^

*Significant differences compared to control group according to ANOVA analysis followed by Tukey's post-test*.

** p < 0.01,

*** *p < 0.001*.

Typical microtubule networks (interphase) and typical bipolar spindles (mitosis) were observed in the MCF-7 control cultures (Figure 3C). By contrast, treated cultures frequently exhibited cells with monopolar mitotic spindles, and multinucleated cells (Figures 3D,E, Supplementary Material Video 1). Cells with asymmetric bipolar spindles were also observed but no significant differences were seen (Figure 3E). Considering previous data obtained by confocal microscopy, we evaluated the mRNA expression levels of the *AURKB, PLK1* and *CDKN1A* genes since these genes encode critical regulator proteins involved with proliferation inhibition and mitosis progression. We observed that calein C significantly reduced the relative mRNA abundance of *PLK1* and *AURKB* and increased the expression of *CDKN1A* (p21) (Figure 3F).

**Figure 4:**
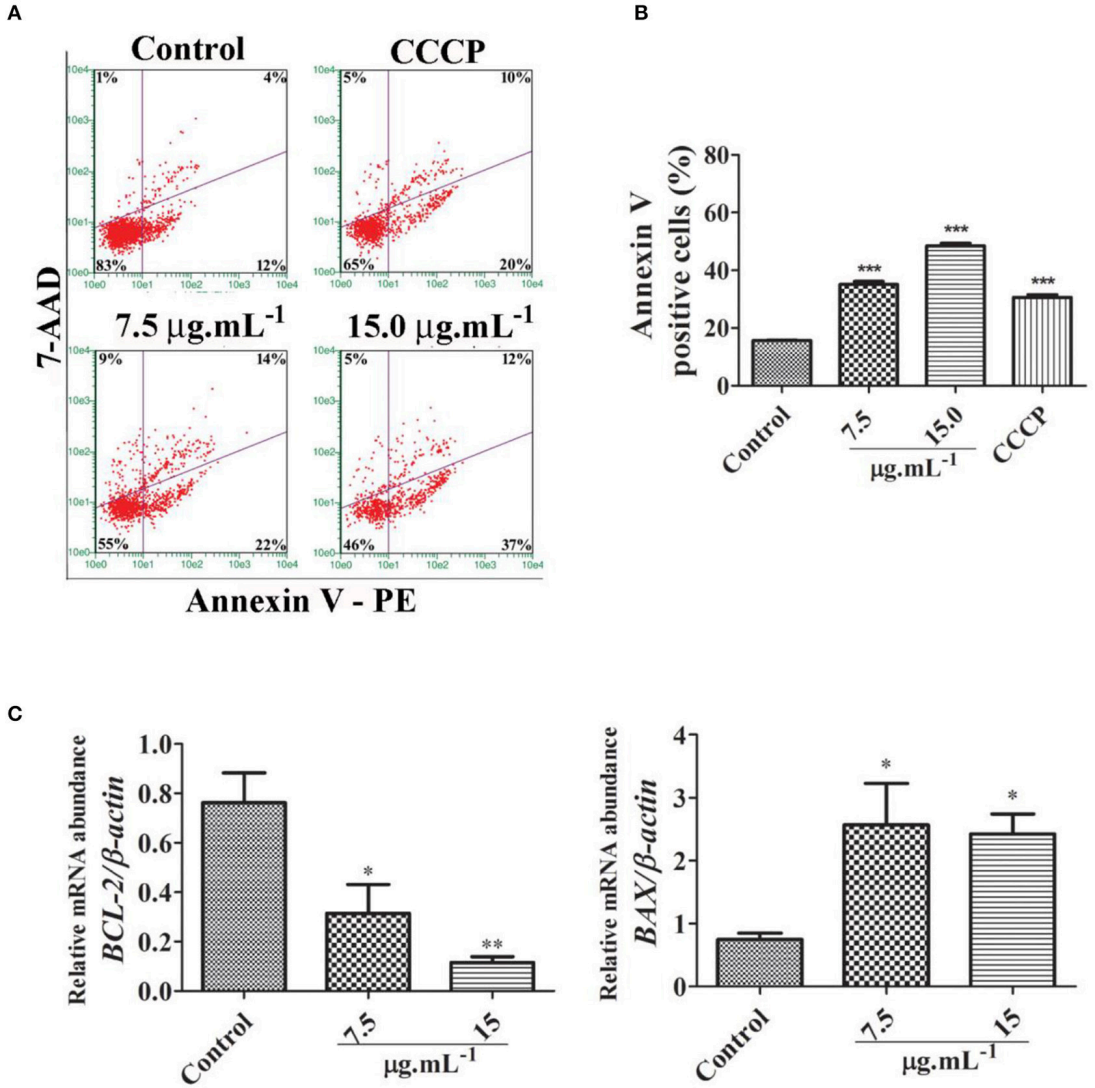
**(A)** Representative dot plots from the annexin V/7-AAD assay performed 24 h after treatment with calein C. Viable cells (lower left quadrants), early apoptotic cells (lower right quadrants), late apoptotic cells (upper right quadrants), and necrotic cells (upper left quadrants). Carbonyl cyanide m-chlorophenylhydrazone (CCCP) was used as a positive control once it reduces ΔΨm. **(B)** Quantitative analysis performed on the annexin V assay data. Data represent the mean ± SD from 3 independent experiments. **(C)** Relative mRNA abundance of apoptotic genes determined by real-time PCR after 24 h of treatment using β*-actin* as a housekeeping gene. Data represent the mean ± SEM from 4 independent experiments. *p < 0.05 and **p < 0.01 according to ANOVA followed by Tukey's post-test.

Further, we evidenced by annexin V assay that there was a significant increase in the number of apoptotic cells in samples treated with calein C compared to the control group (Figures 4A,B). In addition, mRNA abundance of *BCL2* was reduced while the expression level of *BAX* was increased by calein C treatment (Figure 4C).

## Discussion

The structure of calein C as isolated substance was determined based on the spectrometric analyses (NMR and MS). For the definition of the relative positions of the acetate and methacrylate groups, 2D NMR spectra (HMBC and HSQC) were performed. In the spectrum obtained in CDCl_3_ the H-8 and H-9 signals appeared in δ_H_ 5.61 as a singlet, with an overlap of these signals which made the definition of the acetate methacrylate positions doubtful. In this way a benzene-*d*_6_ spectrum was recorded, assigning the signals H-8/C-8 at δ_H_ 6.03/δ_C_ 73.8 and H-9/C-9 at δ_H_ 5.72 /δ_C_ 74.6 in the HSQC spectrum. In addition in HMBC spectrum H-8 and H-9 signals were correlated with each carbonyl: H-9 with δ_C_ 169.8 (acetate group) and H-8 with δ_C_ 165.2 (methacrylate group) confirming the positions of the acetate and the methylacrylate groups.

Calein C, also named caleurticolide acetate, has been isolated since late 1970s and was previously isolated from *C. urticifolia* (Bohlmann and Jakupovic, 1979). This substance was also described in *C. zacatechichi* but with acetate and methacrylate groups being interchangeable (Herz and Kumar, 1980). Additionally (Ferreira et al., 1980) described an isomer of calein C, arucanolide as the major constituent of *C. pinnatifida*. However cconsidering that arucanolide was described from the same plant, we suggested that the structure of the work of (Ferreira et al., 1980) should be reviewed since when it was reported, no 2D NMR experiments were performed to verify the correct position of the acetate and methacrylate groups.

Previous data have shown a high level of cytotoxic activity by calein C on cancer cells derived from malignant solid tumors, including MCF-7. Thus, we initially aimed to compare the cytotoxic activity of calein C on three breast cancer cell lines (MCF-7, ER+; Hs578T, TN, and MDA-MB-231, TN). We found that MCF-7 cells were more responsive than Hs578T or MDA-MB-231 cells to the treatment with calein C. In addition, calein C displayed a good selectivity against MCF-7 cells. These preliminary data led us to choose MCF-7 cells for further investigation, especially since there is no report in the literature of the possible mechanisms underlying the cytotoxic activity of calein C on breast cancer cells.

In the next step, we evidenced that calein C significantly reduced clonogenic capacity of MCF-7 cells demonstrating its ability to inhibit MCF-7 proliferation by prolonged period. These findings indicates a promising antitumor potential of calein C once sustained proliferative behavior is a hallmark of cancer cells and contributes to tumor progression and metastasis (Hanahan and Weinberg, 2011). Further, we demonstrated by flow cytometry that calein C induces cell cycle arrest at G2/M. To date, there has been no report concerning the negative influence of calein C on cell cycle progression. It has only been reported that arucanolide, a calein C analog, induces an increase in a sub-G1 population in U118 (glioblastoma) and HeLa cell cultures (Nakagawa et al., 2005; Marchetti et al., 2012).

We further investigated whether calein C inhibits the G2/M transition or the M-phase progression once cell cycle analysis does not allow the distinction of cells in G2- or M-phase. Thus, we determined the frequency of mitosis and evaluated phospho-Histone H3 (Ser10) expression levels, a marker of mitosis (Lapenna and Giordano, 2009). We found that calein C treatment at 7.5 μg mL^−1^ inhibits mitosis progression, inducing the accumulation of cells in prophase/prometaphase and metaphase. These findings were reinforced by data from immunoblot that showed increased expression of p-Histone H3 levels in samples treated with the same concentration. We did not observe changes in the cyclin B1 expression profile following calein C treatment in tested experimental conditions. There is no report in the literature concerning the antimitotic activity of calein C on tumor cells, especially on breast cancer cells; however, studies have demonstrated an antimitotic effect of some germacranolide sesquiterpenes on multiple myeloma, prostate and cervical cancer cells (Liu et al., 2011; Robles et al., 2015).

Based on previous results, we investigated possible molecular targets of calein C. Successful cell division requires the activity of nuclear kinase proteins, including aurora kinases (aurora A and aurora B) and Polo-like kinase 1 (PLK-1), which phosphorylate a myriad of centrosomal/mitotic protein substrates to insure the fidelity of mitotic progression (Li and Li, 2006; Joukov and Nicolo, 2018). Aurora B (AURB) plays an important role in chromosome and microtubule interactions, spindle stability, and cytokinesis (Carmena and Earnshaw, 2003; Carmena et al., 2009). PLK-1 regulates centrosome separation, spindly assembly, chromosome alignment, and cytokinesis (Vicente and Wordeman, 2015; Otto and Sicinski, 2017). In the current study, we demonstrated that calein C effectively reduced both *AURB* and *PLK1* mRNA abundance in MCF-7 cells. At the same time, *CDKN1A* (p21) mRNA abundance was increased by the calein C treatment. Our findings indicate that calein C induces mitosis arrest, at least in part, due to its ability to modulate Aurora B, PLK-1, and p21 expression. These data are reported for the first time and highlight the antitumor potential of calein C. Aurora kinases (A and B) and PLK-1 are overexpressed in some types of cancer, including breast cancer (Wolf et al., 2000; Weichert et al., 2005; Li and Li, 2006). A study conducted by Donizy et al. (2016) demonstrated that PLK-1 displays an important role in breast cancer progression. Moreover, it was demonstrated by Larsen et al. (2015) that selective inhibition of AURB may be useful to overcome resistance to anti-estrogen therapy.

We associated the downregulation of *PLK-1* and *AURB* induced by calein C to an increase in mitotic cells with monopolar spindles and multinucleated cells. PLK-1 is required for centrosome maturation and the accurate assembly of the bipolar spindle (Strebhardt and Ullrich, 2006) and its inhibition may lead to mitotic arrest by spindle checkpoint activation and subsequent apoptosis (Schöffski, 2009). Similarly, AURB inhibition induces disturbances in the mitotic spindle and chromosome alignment with consequent mitotic arrest and induction of apoptosis (Chopra et al., 2016). Different mechanisms are related to apoptosis induction via AURB inhibition; however, it has been reported that prolonged mitotic arrest may lead to mitotic slippage that, in turn, contributes to the occurrence of multinucleated cells that subsequently undergo apoptosis (Orth et al., 2011, 2012). We will further investigate whether this mechanism is related to the pro-apoptotic activity of calein C on MCF-7 cells. However, we have demonstrated that calein C effectively reduces the *BCL2/BAX* ratio and that it displays great pro-apoptotic activity.

## Conclusion

In the present study, a sesquiterpene lactone, calein C, was isolated from *Calea pinnatifida*, and its chemical structure was characterized by spectroscopic and spectrometric analysis. We demonstrated that this substance effectively inhibited the proliferation of MCF-7 cells, an estrogen-positive breast cancer cell line, through mitotic arrest. The antimitotic effect of calein C was associated with its capacity for reducing *AURB* and *PLK-1* expression levels. The pro-apoptotic activity of calein C was evident in this study due to its ability to reduce the *BCL-2/BAX* ratio. Therefore, calein C is a promising antimitotic agent that should be considered for further *in vivo* studies.

## Authors contributions

LC, MI, PS: project design; LC, MI, RH, GF-S, and PS: experimental work, data collection and evaluation; LC, MI, and PS: literature search and manuscript preparation; MI and PS: funding acquisition; LC, MI, RH, GF-S, and PS: investigation; MF and PS: spectral analysis and interpretations; PS: project administration; LC, MI, and PS: writing–original draft; LC, MI, and PS: Writing–review& editing.

### Conflict of interest statement

The authors declare that the research was conducted in the absence of any commercial or financial relationships that could be construed as a potential conflict of interest.
