# Supplementary material for: Calein C, a Sesquiterpene Lactone Isolated From Calea Pinnatifida (Asteraceae), Inhibits Mitotic Progression and Induces Apoptosis in MCF-7 Cells
Source: Front Pharmacol. 2018 Oct 18;9:1191. doi: 10.3389/fphar.2018.01191 (PMC6201056; doi:10.3389/fphar.2018.01191)
Supplement: Supplementary file 2 [file Data_Sheet_1.pdf]

Calein C, a sesquiterpene lactone isolated from *Calea pinnatifida* (Asteraceae), inhibits mitosis progression and induces apoptosis in estrogen positive breast cancer cells

Lhaís Araújo Caldas [a], ***Marisa Ionta*** [b], ***Renato O. Horvath*** [b], ***Guilherme Álvaro Ferreira da Silva*** [b], ***Marcelo J. P. Ferreira*** [c], ***Patricia Sartorelli*** [a,*]

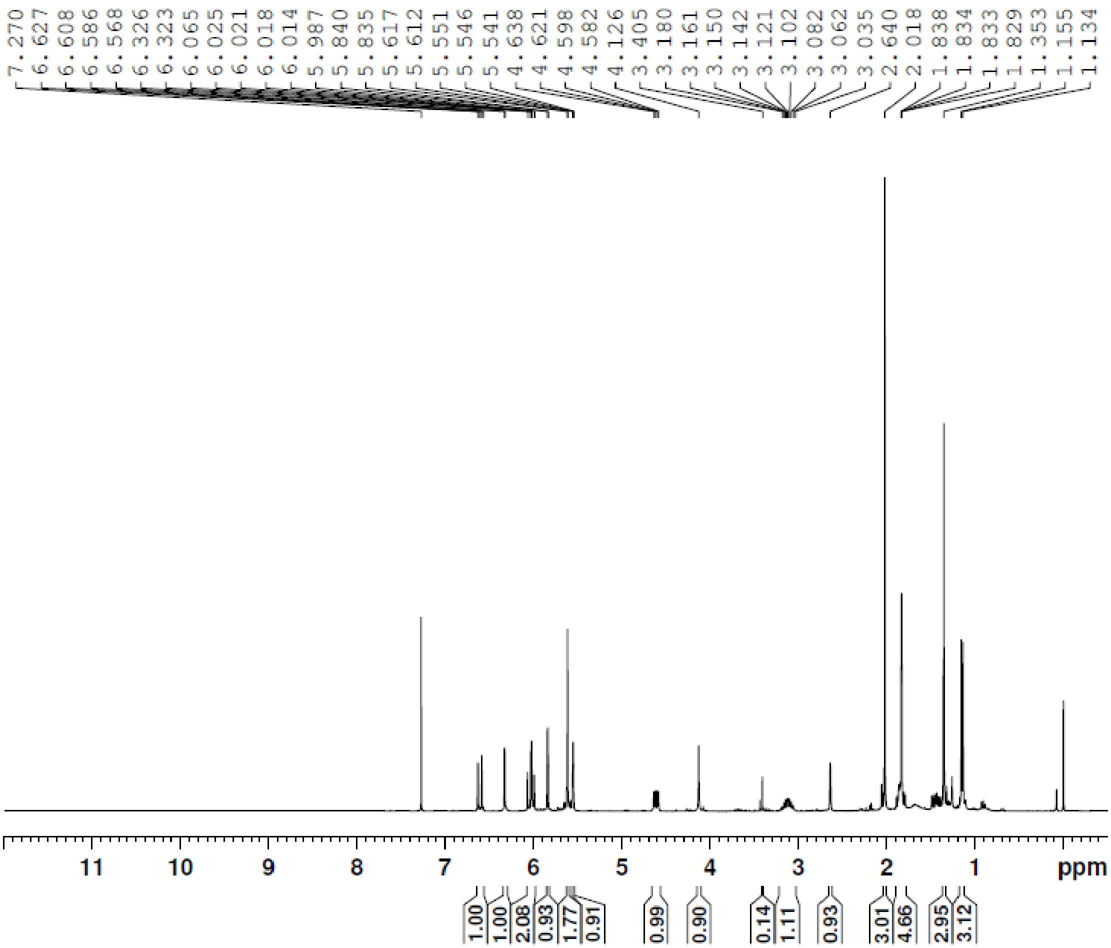

Figure S1(a): ^{1}H NMR spectrum (300 MHz; $CDCl_3$) of *calein C* (**1**)

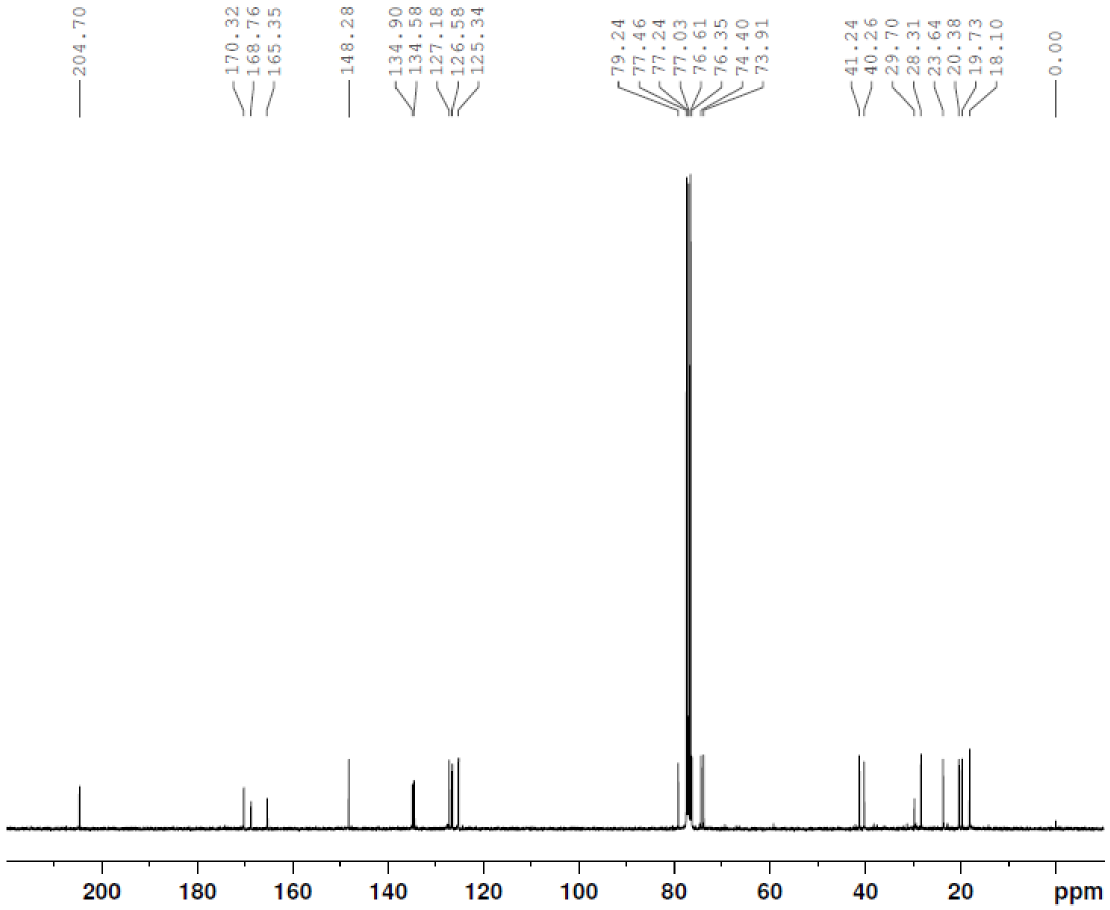

Figure S1(b): ^{13}C NMR spectrum (75 MHz; $CDCl_3$) of *calein C* (**1**)

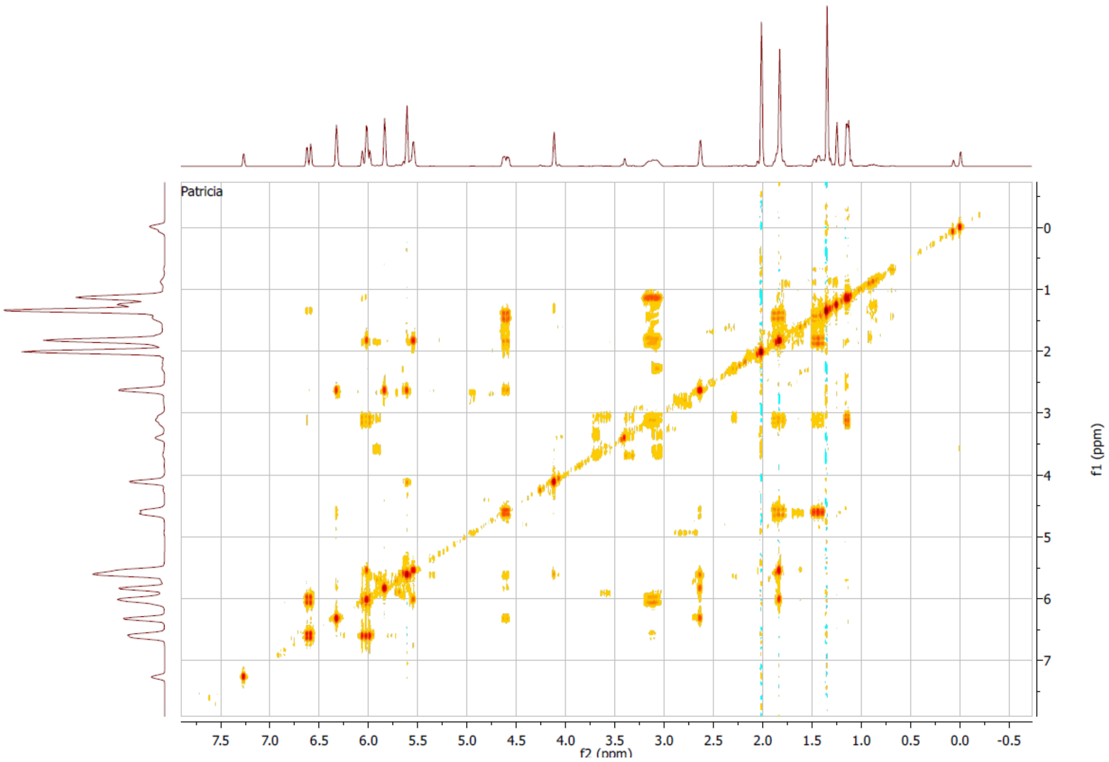

Figure S1(c): COSY spectra (300 MHz, $CDCl_3$) of *Calein C* (**1**)

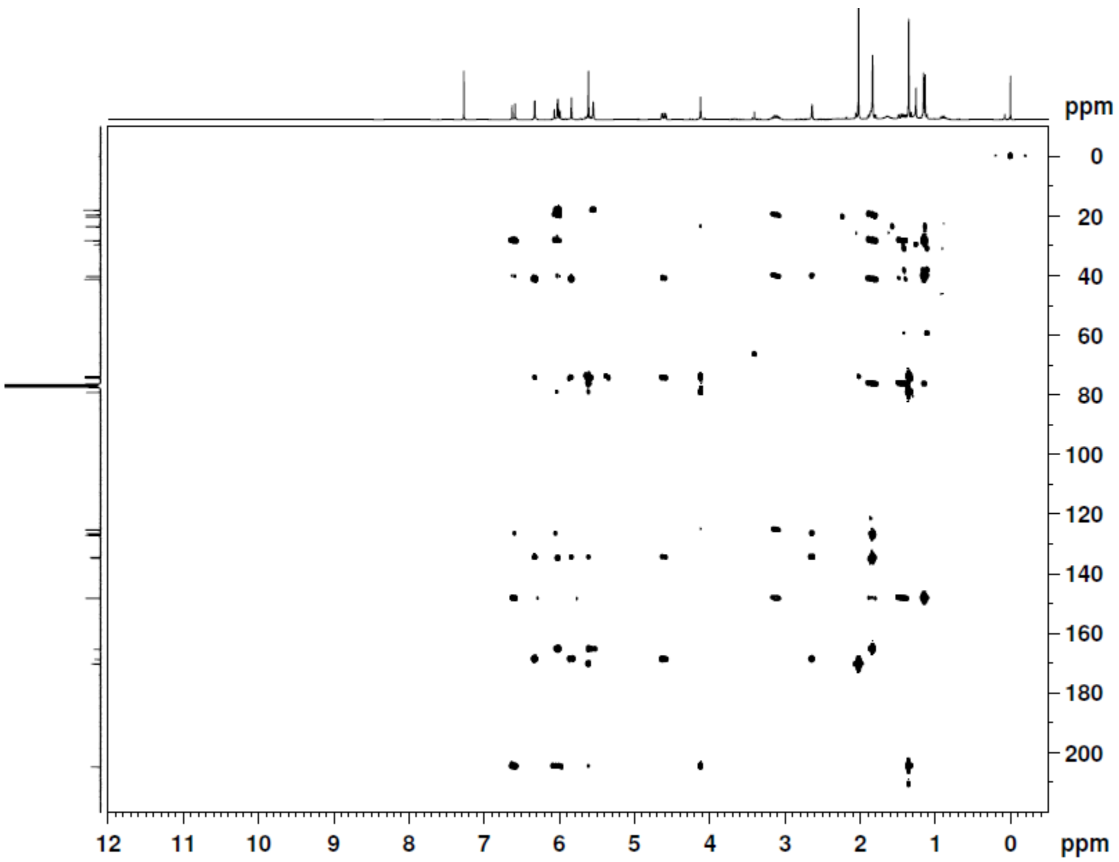

Figure S1(d): HMBC spectra (300 MHz, $CDCl_3$) of *calein C* (**1**)

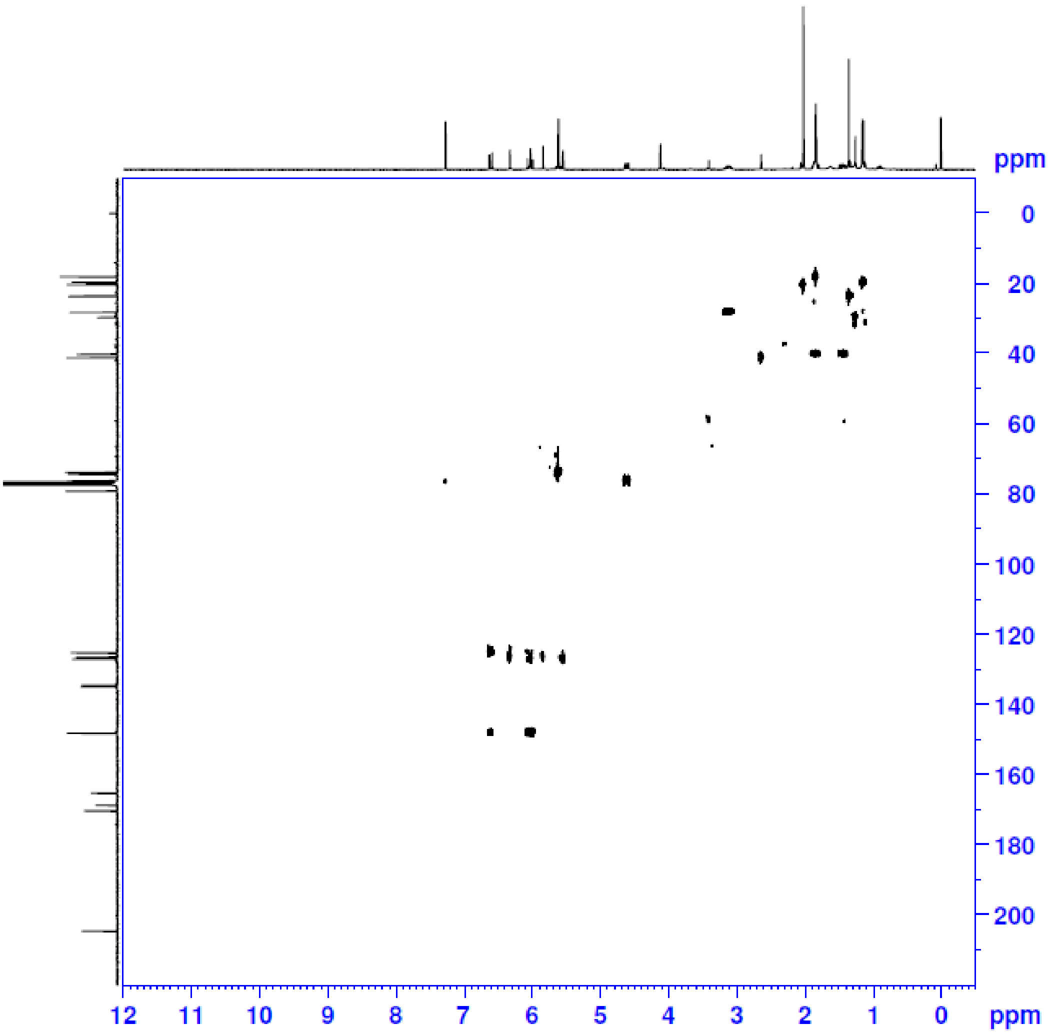

Figure S1(e): HSQC spectra (300 MHz, $CDCl_3$) of *calein C* (**1**)

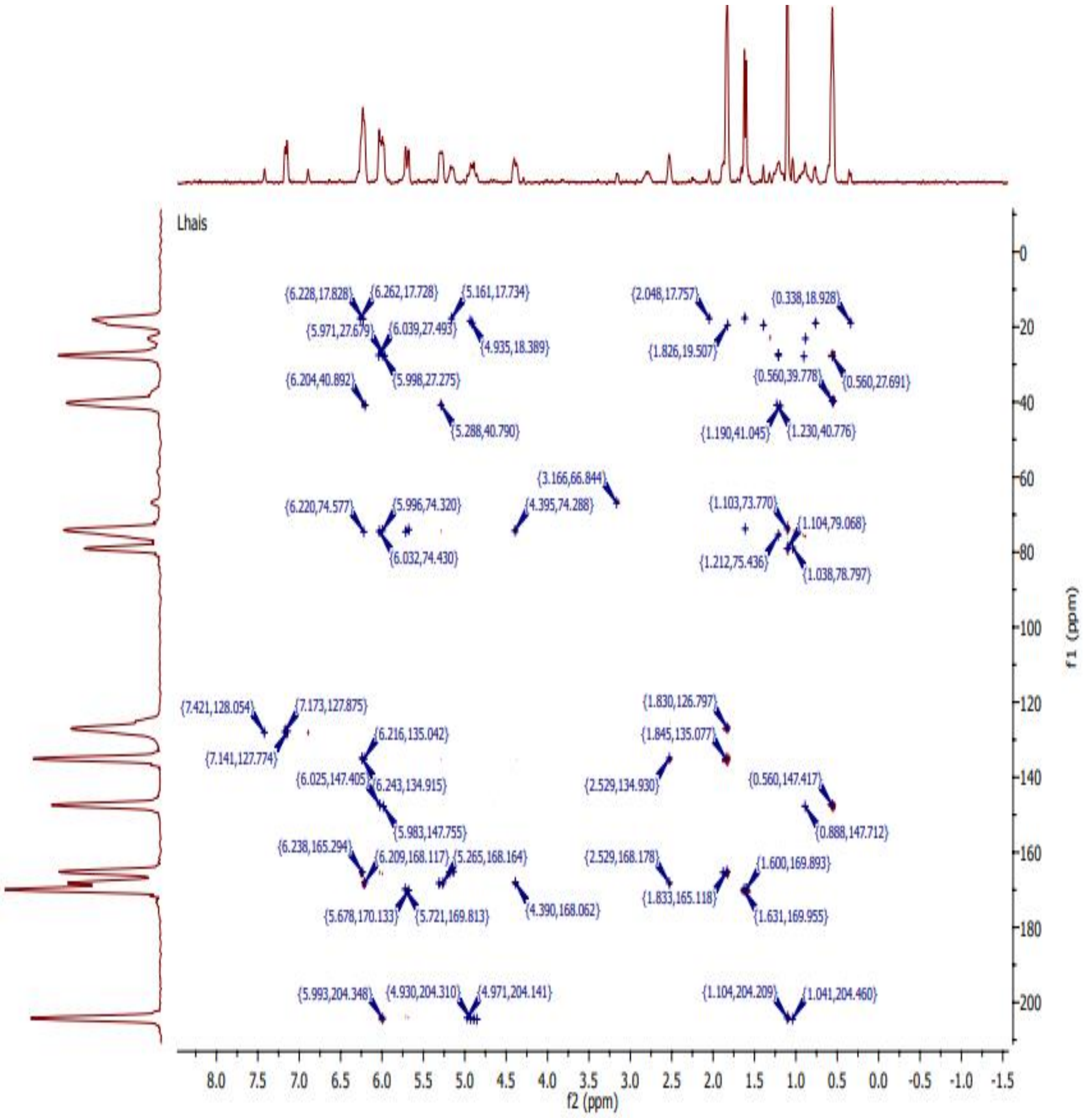

Figure S1(f): HMBC spectra (300 MHz, benzene-d_6) of *calein C* (**1**)
